# Statistical and computational methods for enabling the clinical and translational application of spatial transcriptomics

**DOI:** 10.1002/ctm2.70119

**Published:** 2024-12-07

**Authors:** Peijun Wu, Xiang Zhou

**Affiliations:** ^1^ Department of Biostatistics University of Michigan Ann Arbor Michigan USA; ^2^ Center for Statistical Genetics University of Michigan Ann Arbor Michigan USA

**Keywords:** Computational Methods, Clinical Applications, Spatial Transcriptomics, Statistical Methods

## 1 SPATIAL TRANSCRIPTOMICS AND ITS CLINICAL RELEVANCE

Spatially resolved transcriptomics (SRT) is a collection of innovative genomic technologies that enable gene expression profiling within tissues while preserving spatial context.^1^ These technologies include imaging‐based methods, such as single‐molecule in situ hybridization (e.g. MERFISH, SeqFISH+, MERSCOPE, CosMx and 10x Xenium) and in situ sequencing (e.g. STARmap and Ex‐seq), which typically achieve cellular or subcellular spatial resolution, as well as sequencing‐based methods that offer either spot resolution (e.g. spatial transcriptomics, 10x Visium and Slide‐seq), capturing mixtures of cells with heterogeneous cell types, or higher cellular or subcellular resolution (e.g. seq‐scope, VisiumHD, stereo‐seq and Open‐ST). Together, these technologies transformed the study of tissue biology, providing unprecedented insights into the transcriptomic and cellular landscapes of complex tissues.

SRT has the potential to revolutionize our understanding of the molecular mechanisms driving disease progression and facilitate the identification of diagnostic biomarkers, thus presenting transformative potential in clinical and translational applications. By capturing gene expression patterns across the entire tissue, SRT enables precise identification of transcripts, cell types and tissue areas that underlie disease aetiology, facilitates refined patient stratification, and supports the development of personalized treatments. For example, SRT has been instrumental in distinguishing tumour cells from their surrounding microenvironment, revealing pathways involved in tumour invasion and metastasis, and highlighting the spatial and transcriptomic heterogeneity across cancer subtypes, thus directly informing clinical oncology by advancing diagnostics and guiding targeted therapies.^2^, ^3^, ^4^ Additionally, spatial patterns of drug resistance or immune cell infiltration observed in patient tissue samples can inform treatment strategies and support individualized clinical decision‐making.^4^, ^5^ Overall, SRT promises to bridge fundamental research with clinical applications, advancing our capacity to diagnose, treat and monitor complex diseases with unprecedented precision.

**FIGURE 1:**
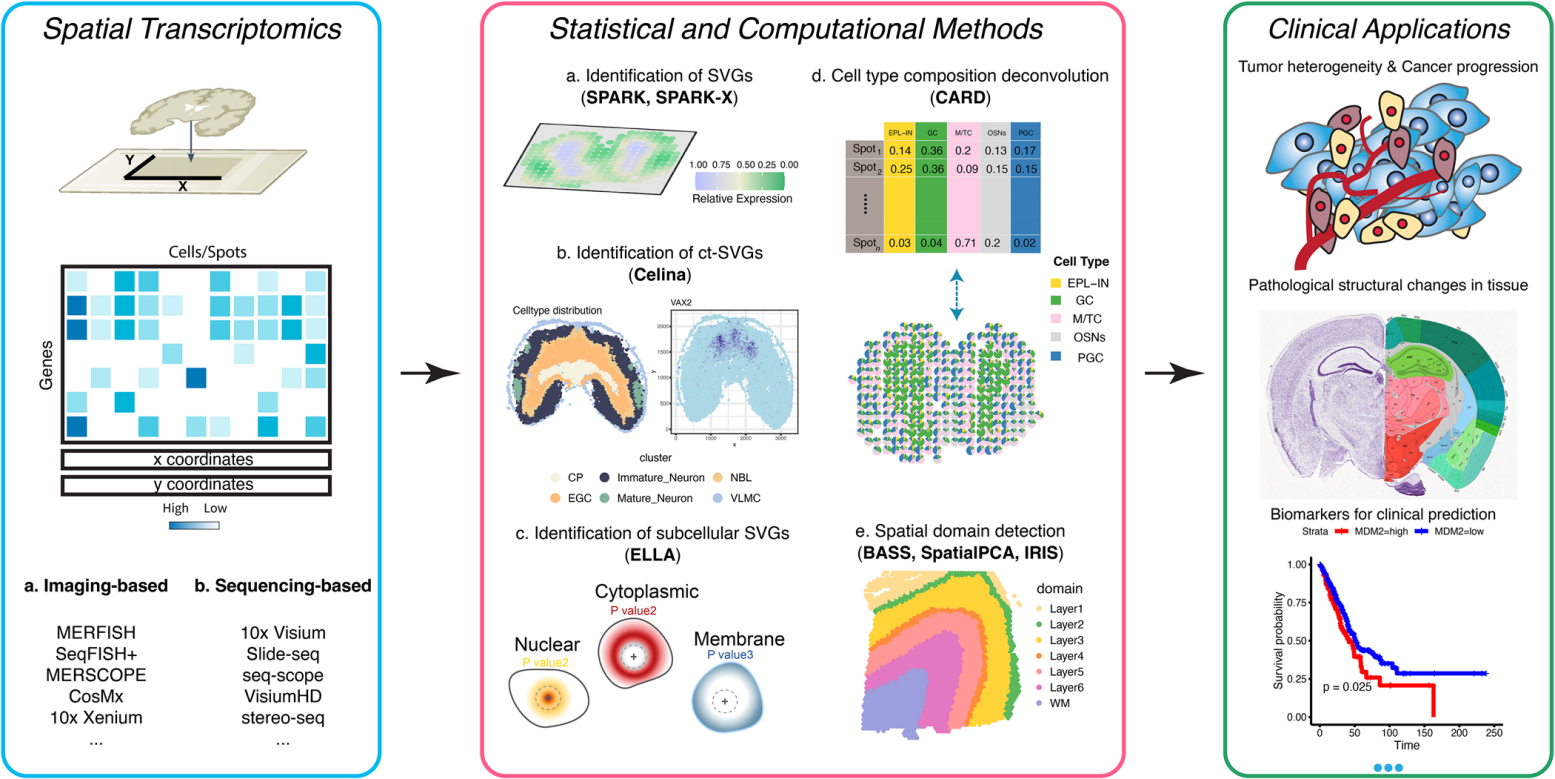
Spatially resolved transcriptomics (SRT) encompasses a set of innovative genomic technologies that enable gene expression profiling within tissues while preserving spatial context. Left panel: SRT data include a gene expression matrix that contains expression measurements across spatial locations on the tissue, along with corresponding x/y coordinates. SRT data can be obtained from either imaging‐based technologies or sequencing‐based technologies. Middle panel: These SRT data are analyzed using a range of recently developed statistical and computational methods that facilitate the detection of spatially variable genes (SVGs)—whether overall, cell type‐specific or subcellular. Additionally, these methods support the deconvolution of cell type composition across tissue locations, detection of spatial domains, and inference of pseudo‐time trajectories on the tissue. Right panel: These statistical and computational analyses are essential to advancing SRT studies toward clinical and translational applications, enabling the study of tissue heterogeneity in cell type composition and gene expression, the mapping of tissue architecture atlases and the identification of biomarkers for clinical diagnosis. (Note that the mouse brain anatomic reference on the right panel is obtained from the Allen Mouse Brain Atlas^10^).

Statistical and computational methods for SRT analysis are essential for harnessing its potential towards clinical applications (Figure 1). In this context, we briefly discuss several important analytical tasks that facilitate the use of SRT in clinical and translational medicine, along with the statistical and computational methods that enable these analyses.

## 2 IDENTIFICATION OF SPATIALLY VARIABLE GENES, CELL‐TYPE‐SPECIFIC SPATIALLY VARIABLE GENES AND SUBCELLULAR SPATIALLY VARIABLE GENES

Spatially variable genes (SVGs), also known as spatially expressed genes, exhibit specific spatial expression patterns across tissue sections. These genes capture transcriptomic signatures that reflect the topographical organization of complex tissues, contributing to the spatial arrangement of tissue functions.^6^, ^7^ Common methods for identifying SVGs include SPARK^6^ and SPARK‐X.^7^ SPARK uses an over‐dispersed Poisson model with a generalized linear spatial component and incorporates multiple spatial kernels to directly model count data. In contrast, SPARK‐X provides a non‐parametric approach using a robust covariance test for sparse count data, delivering significant computational efficiency. Both methods ensure type I error control and high power, with SPARK offering particularly high statistical power, while SPARK‐X achieves notable speed improvements. Both SPARK and SPARK‐X have been widely applied in SRT data analysis, uncovering critical biomarkers essential for clinical diagnosis, characterizing disease subtypes, and monitoring disease progression across various conditions.

Many identified SVGs serve as cell type markers, displaying spatial expression patterns that reflect the distribution of distinct cell types. However, a significant subset of SVGs, known as cell‐type‐specific SVGs (ct‐SVGs), shows diverse spatial expression patterns within specific cell types.^5^ SPARK‐X analysis has demonstrated that approximately half of the detected SVGs are ct‐SVGs, highlighting their role in revealing spatial transcriptomic heterogeneity within individual cell types.^7^ These ct‐SVGs are critical for understanding transcriptomic mechanisms underlying cellular heterogeneity—how cells of the same type can exhibit different functional states, respond to local microenvironments, engage in unique signalling pathways, and undergo dynamic regulatory processes related to cell state transitions during development and disease progression. An important method for identifying ct‐SVGs is Celina,^5^ which employs a spatially varying coefficient model that captures gene expression patterns within specific cell types, ensuring robust type I error control and high power. ct‐SVGs detected by Celina have been shown to uncover complex developmental processes, serve as valuable biomarkers for disease diagnostics, and provide insights into cell differentiation, tissue development and organization, and disease progression.

As SRT technologies advance to achieve subcellular resolution, the detection of subcellular SVGs that exhibit spatial variation within individual cells becomes possible. Currently, ELLA^8^ is the only method designed specifically to model subcellular mRNA localization and detect subcellular SVGs. ELLA establishes a unified cellular coordinate system and utilizes a nonhomogeneous Poisson process to ensure type I error control and high power, enabling the identification of subcellular gene expression patterns and the subcellular mechanisms underlying disease aetiology. Subcellular SVGs detected by ELLA are crucial for understanding transcriptomic changes at the subcellular level during disease onset and progression, supporting advancements in clinical diagnosis and treatment across various diseases.

## 3 SPATIAL DOMAIN DETECTION

Tissues are complex ecosystems composed of spatially organized and functionally distinct domains and microenvironments, each characterized by unique cell type compositions and transcriptomic heterogeneity. These localized domains coordinate cellular functions such as development, communication, and repair. Detecting spatial domains thus reveals critical insights into tissue organization and structural changes underlying various diseases.

Common methods for spatial domain detection include BASS,^9^ SpatialPCA^4^ and IRIS,^3^ each capable of handling multiple tissue sections. BASS employs a Bayesian hierarchical model to conduct multi‐scale transcriptomic analysis, facilitating joint cell type clustering and spatial domain detection. SpatialPCA models spatial correlation across tissue locations to produce a low‐dimensional embedding that preserves neighbourhood similarities. This spatially informed embedding enables domain detection, pseudo‐time trajectory inference, and imputation of gene expression for unmeasured spatial locations, resulting in refined spatial maps that go beyond the original study resolution. IRIS, a highly scalable method, is currently the only available tool for detecting spatial domains on large‐scale platforms like Stereo‐seq and 10x Xenium. IRIS models cell‐type compositional heterogeneity across locations, using single‐cell RNA sequencing data for reference‐informed detection of biologically meaningful spatial domains.

These spatial domain detection methods enable comprehensive analysis of tissue structures and microenvironments. By characterizing the spatial distributions of distinct cell types across domains, they allow for trajectory analysis to explore development and disease progression. For example, these methods have been used to characterize disease tissue heterogeneity by distinguishing tissue subregions and detecting tertiary lymphoid structures that influence transcriptomic transitions during tumorigenesis. Identified regions further enable the discovery of key molecular signatures associated with specific tissue areas and microenvironments, such as tumour‐suppressor genes and immune cell subsets across tumour‐adjacent and non‐tumour‐adjacent stroma. Additionally, these approaches detect pseudo‐time‐associated genes enriched in immune response pathways, highlighting their value in studying cancer progression and metastasis. Collectively, these methods facilitate clinical diagnosis and potential treatment planning.

## 4 CELL TYPE COMPOSITION DECONVOLUTION

Many commonly applied SRT technologies, particularly sequencing‐based methods like 10x Visium, often have limited spatial resolution, measuring tissue locations that encompass only a few to several dozen cells, which may belong to distinct cell types. Cell‐type deconvolution is a crucial analysis that aims to estimate the cell type composition at each measured location on the tissue, thereby helping to disentangle the spatial localization of cell types and facilitating a better understanding of complex tissue architecture.

CARD^2^ is a widely used method for cell‐type deconvolution. It utilizes non‐negative matrix factorization to model spatial correlations in cell‐type composition through a conditional autoregressive model. CARD can perform both reference‐based and reference‐free cell‐type deconvolution, depending on the availability of single‐cell RNA sequencing reference data. Importantly, CARD is also capable of constructing a refined spatial tissue map of cell type or gene expression with a resolution that can exceed that measured in the original study. CARD's ability to uncover the spatial distribution of cell types and molecular markers provides valuable insights into the identification of cell types associated with disease onset and progression, thus facilitating clinical applications.

## 5 CONCLUSIONS

Statistical and computational methods are essential for harnessing the potential of SRT to transform tissue biology, deepen our understanding of tissue structure and disease mechanisms and advance clinical and translational medicine.

## AUTHOR CONTRIBUTIONS

**Xiang Zhou**: conceived the idea and provided funding support. **Peijun Wu and Xiang Zhou**: wrote the manuscript.

## CONFLICT OF INTEREST STATEMENT

The authors declare no conflict of interest.
